# High Expression of *hTERT* and Stemness Genes in BORIS/CTCFL Positive Cells Isolated from Embryonic Cancer Cells

**DOI:** 10.1371/journal.pone.0109921

**Published:** 2014-10-03

**Authors:** Loredana Alberti, Stéphanie Renaud, Lorena Losi, Serge Leyvraz, Jean Benhattar

**Affiliations:** 1 Institute of Pathology, Lausanne University Hospital, Lausanne, Switzerland; 2 Institute of Biotechnology, University of Lausanne, Lausanne, Switzerland; 3 Department of Life Sciences, University of Modena and Reggio Emilia, Modena, Italy; 4 Department of Oncology, Lausanne University Hospital, Lausanne, Switzerland; 5 Biopath Lab, Lausanne, Switzerland; University of Newcastle, United Kingdom

## Abstract

BORIS/CTCFL is a member of cancer testis antigen family normally expressed in germ cells. In tumors, it is aberrantly expressed although its functions are not completely well-defined. To better understand the functions of BORIS in cancer, we selected the embryonic cancer cells as a model. Using a molecular beacon, which specifically targets *BORIS* mRNA, we demonstrated that BORIS positive cells are a small subpopulation of tumor cells (3–5% of total). The BORIS-positive cells isolated using BORIS-molecular beacon, expressed higher telomerase *hTERT*, stem cell (*NANOG, OCT4, SOX2*) and cancer stem cell marker genes (*CD44* and *ALDH1*) compared to the BORIS-negative tumor cells. In order to define the functional role of BORIS, stable BORIS-depleted embryonic cancer cells were generated. *BORIS* silencing strongly down-regulated the expression of *hTERT*, stem cell and cancer stem cell marker genes. Moreover, the BORIS knockdown increased cellular senescence in embryonic cancer cells, revealing a putative role of BORIS in the senescence biological program. Our data indicate an association of BORIS expressing cells subpopulation with the expression of stemness genes, highlighting the critical role played by BORIS in embryonic neoplastic disease.

## Introduction

Brother of the regulator of imprinting sites (BORIS) also designed as CTCFL, CCCTC-binding factor-like, is a DNA-binding protein with functions in cancer not fully understood. CTCF is a highly conserved, ubiquitously expressed, multifunctional chromatin factor that plays a role as a tumor suppressor gene [1]–[3]. BORIS is a mammalian paralog of CTCF, they share the same 11 zinc-finger domain but differ at N- and C- termini. Within this zinc-finger domain, BORIS and CTCF exhibit 70% of homology [4]. In normal tissues, *BORIS* expression is restricted to germ cells, where it is involved in epigenetic reprogramming [4], [5]. *BORIS* is expressed in spermatocytes during male germ line development, apparently in absence of CTCF [4]. In tumors, BORIS is aberrantly expressed and its transcription was detected at different levels in several cancer cell lines and in primary tumors [6]. Due to its restricted expression in normal germinal tissues and its re-expression in a wide variety of tumors, BORIS belongs to cancer testis antigen (CTA) family. It has been shown that BORIS induced expression of other CTA genes, as MAGE-A1, NY-ESO-1 [7], [8] and SPANX [9] but not in all tumors [10], [11]. In addition, we previously showed that BORIS activated *hTERT* expression by binding to the first exon of the *hTERT* telomerase gene in embryonic and ovarian tumor cells [12]. Furthermore, in studies of exogenous BORIS expression in normal BORIS-negative cells, we demonstrated that these transfected cells exhibited high levels of *hTERT* mRNA [12]. All these results revealed an important role of BORIS in the immortalization process during tumorigenesis. Interestingly, current reports show a correlation between *hTERT* expression and stem cell-like properties [13]–[17]. Further investigations regarding the correlation between BORIS functions and the main roles of hTERT in the immortalization and stemness properties have to be performed. Another question not yet clearly answered is how many cells, within a tumor cell line, express *BORIS*. In this paper, we address these questions. For this purpose, we selected to use the molecular beacon (MB) imaging technology, as it is an approved method to detect and also to visualize mRNA expression [18]. MBs are oligonucleotides structured as stem-loop hairpin with a fluorescence quencher at one end and, at the opposite end, a fluorescent dye also called fluorophore. Due to their specific structure, MBs emit fluorescence signals only when binding to their targets. To explore the frequency of BORIS-positive cells within tumor cell lines, we first designed a *BORIS* mRNA targeting MB, and then we analyzed *BORIS* expression in human embryonic and ovarian tumor cell lines, respectively NCCIT and OVCAR3. After verifying that BORIS-MB enable FACS sorting of BORIS-positive cells, we showed that the isolated BORIS-positive cells expressed higher mRNA level of *hTERT* and stemness genes compared to BORIS-negative and non-sorted NCCIT cells. We further confirmed this result by *BORIS* silencing studies. Moreover, we showed that BORIS protects from senescence process. Altogether, our data confirm a direct role of BORIS in embryonic neoplastic disease.

## Materials and Methods

### Cells

The human cell lines (BJ, foreskin fibroblast; HeLa, cervical adenocarcinoma; NCCIT, embryonic carcinoma; OVCAR3, ovary carcinoma) were purchased from the American Type Culture Collection (ATCC). The cells were cultured at 37°C with 5% CO_2_ either in Dulbecco's modified Eagle's medium (DMEM; Gibco, Invitrogen) for HeLa and BJ cells, or in RPMI-1640 medium (Gibco, Invitrogen) for NCCIT and OVCAR3, supplemented with 10% of heat inactivated fetal bovine serum (Invitrogen) and 1% of Penicillin-Streptomycin (Gibco, Invitrogen).

### Molecular beacon (MB) design

Sequences of BORIS-MB1 and BORIS-MB2 were designed using Beacon Designer (Premier Biosoft). *BORIS* mRNA secondary structures were predicted using mFOLD software (mFOLD, http://www.bioinfo.rpi.edu/applications/mfold/) and specificity was determined by BLAST search (NCBI). The target sequence of BORIS-MB1 is located on exon 2 and that of BORIS-MB2 is located on exon 11 of *BORIS* mRNA. These locations were chosen since they are outside the zinc-finger domain and do not cross-hybridize with the CTCF homology regions. In addition, previous study has shown that the starting and the ending regions of mRNA are the more accessible for MBs hybridization [19]. The RANDOM-MB that was used as negative control does not match with any mammalian sequences [19]. Sequences were the following: BORIS-MB1 5′-CGCTGTCTCTGCACACTCCGTCTTCAGCG-3′; BORIS-MB2 5′-CAGCCATTCCTCTTTGACTCTGGCTG-3′ and RANDOM-MB 5′-CGACGCGACAAGCGCACCGATACGTCG-3′ (underlined bases indicating those complementary to the target sequences). A fluorophore (Cy3 or ATTO647) was 5′-conjugated and a Black Hole Quencher (BHQ-2) was linked to the 3′-end. The MBs were purchased from Sigma and they were purified by high-pressure liquid chromatography.

### *In vitro* determination of MB specificity

Oligos were designed to be specific of the MBs targets (BORIS-MB1 specific target: 5′-AAGACGGAGTGTGCAGAGAGA-3′; BORIS-MB2 specific target: 5′-CAGCCAGAGTCAAAGAGGAA-3′ and RANDOM-MB specific target: 5′-TATCGGTGCGCTTGTCG-3′). A non-specific oligo was used for the *in vitro* test of the different MBs. This non-specific oligo, 5′-CGATGCCGAACCAATTCTCCAC-3′, corresponds to a transcript variant of CTCF. To test the specificity in solution, 200 nM of MB was mixed or not with 1 µM of oligo in 10 µL of Opti-MEM medium (Invitrogen).

The emission fluorescence profiles were obtained after heating the MB-target oligo mix to a progressive temperature elevation ranging from 15 to 80°C using 1°C steps. Fluorescence signal was acquired at the end of each increasing degree and detected on the Cy3 channel using a Rotor Gene 6000 Real-Time PCR system (Corbett Life Science).

### MB delivery and cell fluorescence imaging

Cells were detached using 0.05% trypsin-EDTA (Invitrogen) and resuspended in serum-free DMEM medium at the concentration of 10^6^ cells/ml. Firstly, Cy3-BORIS-MB or Cy3-RANDOM-MB (200 nM) was incubated at room temperature in presence of 1 µl/ml of Lipofectamine RNAiMAX siRNA transfection reagent (Invitrogen) using Opti-MEM medium. The Lipofectamine RNAiMAX reagent was used as delivery vehicle since in our conditions it gave less background compared to other reagents such as Streptolysin (data not shown). After 10 min, the transfection mix was added to the suspended cells and together incubated for 1 hour at 37°C. Hoechst 33342 (Invitrogen) was added at concentration of 5 µg/mL during the last 10 min of incubation. Then, cells were washed using Phosphate Buffered Saline (PBS, Invitrogen) and resuspended in PBS with 5 mM EDTA. Transfected cells were cytocentrifugated onto glass slide using a cytospin centrifuge and examined under fluorescent microscope (Axioplan2 Imaging, Zeiss). The fluorescence signal of Cy3-coniugated MB was analyzed using the red channel and Hoechst 33342 fluorescence emission was observed under blue channel.

### FACS analysis and sorting using MB

For FACS analysis and cell sorting, we used MBs conjugated with ATTO 647, a dye characterized by its high photostability [20]. Cells were prepared and incubated with MBs as described above (except that Hoechst 33342 was not added) and were directly analyzed using Gallios flow cytometer (Beckman Coulter). At least 10,000 events were collected and analyzed by Kaluza Software. The BORIS-positive and BORIS-negative populations were sorted after exclusion of dead cells by Propidium Iodide (PI) staining using FACSAria I (Becton Dickinson) instrument at the Flow Cytometry Facility of UNIL (University of Lausanne, Switzerland). Ranges of 2×10^4^–9×10^4^ BORIS-positive cells and 2×10^5^–9×10^5^ BORIS-negative cells were sorted.

### BORIS knockdown by inducible shRNA lentiviral system

Stable cell lines with inducible expressing shRNAs targeting human *BORIS* mRNA were generated using the doxycycline-inducible shRNA lentiviral system, pINDUCER [21]. The lentiviral vector pINDUCER11 constitutively expresses the eGFP fluorescent reporter protein, which enables to track cells transduced by the virus. This vector also contains a cassette with a doxycycline-inducible promoter that controls the transcription of a tRFP reporter gene together with the shRNA, which allows detection of cells with doxycycline activated shRNA transcription [21]. Four different shRNAmiR (shRNA) specifically targeting *BORIS*, and not its parolog *CTCF* (BORIS-sh1: 5′-ATTCACCAAGATCAAAGAACTC-3′, BORIS-sh2: 5′-GTTCTCACAGTTTCAAATTCAA-3′, BORIS-sh3: 5′-TTCATCCCGACTGTTTACAAAT-3′, BORIS-sh4: 5′TCCGACAGAAGCAACTTCTAAA-3′) and a control shRNA with scrambled sequence (CTR sh: 5′CAGAGCTAACTCAGATAGTACT3′) were synthetized (Sigma). They were PCR amplified and cloned into the pINDUCER11 backbone using *EcoRI* and *XhoI* restriction enzymes. The sequences of all constructs were verified by sequencing. Lentivirus were generated by co-transfection of the appropriate shRNA constructs along with the packaging vectors (pMD2G-VSVg, pCMV-dR8.74) into HEK-293T cells using FuGENE 6 reagent (Roche Diagnostics) according to the manufacturer's protocol. Viral supernatants were harvested 48 hours after transfection, filtered through a 0.45 µm pore filter, ultracentrifugated for 1.5 hours at 19,500 rpm in a Beckman SW28 rotor and resuspended in RPMI medium. The viral suspension combined with 8 µg/ml polybrene (Sigma) was used to infect target cells (NCCIT). Twenty-four hours post infection the medium was replaced and stably infected cells were eGFP-sorted using FACSAria I instrument (Becton Dickinson) at the Flow Cytometry Facility of UNIL. Induction of shRNA expression was obtained by addition to the medium of 2 µg/ml of doxycycline (Sigma). To maintain the knockdown, doxycycline-containing medium was refreshed every 3 days.

### Ectopic BORIS expression in HeLa cells

The day prior transfection, HeLa cells were seeded at a density of 2×10^5^ cells/well in 12-well/plates. Cells were transfected with 3 µg of the previously described pCMV-BORIS vector [12] using the Lipofectamine 2000 transfection reagent (Invitrogen) following the manufacturer's instructions. Cells were harvested 2 days post-transfection for cell fluorescence imaging.

### Quantitative RT-PCR

Total RNA was isolated using the RNeasy mini kit (Qiagen) including on-column DNAse treatment according to the manufacturer's instructions. RNA concentration was determined using Nanodrop 2000 (Thermo Scientific) and Qubit Fluorescent Technology (Invitrogen).

A major limiting step was the low amount of total RNA isolated from cell sorting. To solve this technical limitation, we applied a method already described and validated [22], [23]. Firstly, 200 ng of total RNA were retrotranscribed using random hexamers and Superscript III reverse transcriptase (Invitrogen) in a final volume of 20 µl. Then 2 µl of cDNA were used for a preamplification reaction consisting on a multiplex PCR made with a mix of primers (Table S1) at 0.1 µM final concentration, 0.5 unit of Platinum Taq DNA Polymerase (Invitrogen), 1X PCR buffer, and 2 mM MgCl_2_ in a total volume of 25 µl.

For preamplification, PCR cycling conditions were: one denaturation step at 95°C for 5 min followed by 15 cycles of amplification (45 sec at 95°C, 30 sec at 60°C, 1 min at 72°C). Finally, for quantitative PCR, the preamplification reaction was 20-fold diluted and 2 µl of this dilution were used as template. Reaction was complemented with 0.5 units Platinum Taq DNA Polymerase (Invitrogen), 1X PCR buffer, 2.5 mM MgCl_2_, 2.5 µM SYTO9 green (Invitrogen) and 0.1 µM of each gene specific primer (Sigma) in a final volume of 20 µl. PCR conditions were: 95°C for 5 min followed by cycles (5 sec at 95°C, 30 sec at 60°C, 45 sec at 72°C). Melting curve analyses were performed at the end of the amplification to check the purity of the amplicons. The PCR products were also loaded on agarose gel to confirm the correct size of amplified products. Cycling and fluorescence acquisition were done in Rotor-Gene 6000 Real-Time PCR system (Corbett Life Science). Data analysis was performed using Rotor-Gene 6000 software. Relative expression levels were determined by ΔΔCt method with expression levels normalized to *GAPDH* level. The PCR efficiency ranged from 94 to 101%, with correlation coefficient (r^2^) ranging from 0.96 to 1.0, for all the amplified target genes. The Ct values of *GAPDH* were roughly the same (Ct = 10.07±0.25) for all the cells used in the quantitative RT-PCR experiments.

The human specific primers (Table S1) were designed using Primer3 (http://bioinfo.ut.ee/primer3-0.4.0/) and OLIGO Primer Analysis software. Specificity was verified using BLAST search (NCBI). The designed primer pairs cross intron-exon boundaries to avoid genomic DNA contamination. BORIS primers were chosen (between exon 8 and 9) based on results previously obtained [24] in order to amplify the most abundant *BORIS* isoforms.

### DNA methylation analysis

DNA was extracted using DNeasy kit (Qiagen). A range of 200 ng (from sorted cells) and 500 ng of DNA was used to bisulfite reaction using EpiTect Bisulfite kit (Qiagen) according to the manufacturer's instructions. The modified DNA was used to amplify a 123 bp fragment of the BORIS promoter.

The methylation assay was designed using the PyroMark Assay Design Software 1.0 (Qiagen). This assay allowed sequencing of 50 bp (from position −968 to −918) inside the promoter B of *BORIS* [25] and included 10 CpG. To perform sequencing, 3 µl of bisulfite treated DNA were first amplified by PCR. Sequences of the PCR primers were: BORIS-pyro Forward 5′ TGGTTTGTGGGTTTTGT 3′ and BORIS-pyro BIO Reverse 5′ CCCTTCACCCCCCCTCTTT 3′. PCR conditions were as follow: 95°C for 5 min; 45 cycles of 95°C for 30 s, 58°C for 15 s and 72°C for 1 min; and a final extension step at 72°C for 10 min. Then, purification and subsequent processing of the biotinylated single-stranded PCR fragment were performed according to the manufacturer's recommendations. Pyrosequencing of this PCR fragment was performed on a PyroMark Q24 instrument using Pyro Gold Q24 Reagents (Qiagen). The pyrosequencing primer (5′ GTGTTGTAGTTTATAGT 3′) was used at a final concentration of 0.3 µM. Resulting data were analyzed and quantified with the PyroMark Q24 software (Qiagen) which calculates the methylation percentage for each CpG site, allowing quantitative comparisons.

### Cell proliferation assay

Cell proliferation was assessed by MTT assay. MTT (3-(4,5-dimethyl-2-thiazol)-2,5-diphenyltetrazolium bromide, Sigma) reagent was used according to the manufacturer's instructions. Briefly, stably infected cells were seeded at a density of 25×10^3^ cells/well in 24-well/plates with doxycycline-containing medium. After 3 days, cells were incubated with MTT reagent (200 µg/ml final concentration) for 3 hours at 37°C. Then, cells were lysed adding isopropanol/HCl for 10 min and the plates were gently shaken for 5 min. Absorbance values were determined using a microplate reader (Synergy Mx, BioTek) at 570 nm. Each experiment was performed in triplicate and 3 independent experiments were conducted.

### Apoptosis analysis

Apoptosis was measured in triplicates using Annexin V Apoptosis Detection Kit (BD bioscience) according to the manufacturer's protocols. Briefly, 5×10^4^ cells/well were seeded in 12-well/plates and were grown in presence of doxycycline until confluence (5–7 days). The floating cells as well as trypsinized cells were collected, washed with PBS and resuspended in 100 µl Binding Buffer. Then, 5 µl of Annexin V-V500 and 5 µl of 7AAD were added and incubated with the cells for 30 min at room temperature. After addition of 400 µl of Binding Buffer the samples were immediately analyzed by Gallios flow cytometer (Beckman Coulter). At least 5×10^4^ events were counted for all samples. The percentage of apoptotic cells was estimated after gating on eGFP and tRFP (transduced and doxycycline-induced, respectively) positive cells.

### Western blot analysis

Whole cell lysates were obtained using RIPA buffer (Sigma) in presence of protease inhibitor cocktail (Sigma) and quantified using the BCA assay (Thermo Scientific). Thirty micrograms of protein were loaded on a 10% SDS-polyacrylamide gel, followed by blotting on a nitrocellulose membrane using a semi-dry transfer apparatus (BIO RAD). Non-specific binding was blocked by overnight incubation in 5% non-fat dried milk in TBS-T buffer (0.1% Tween 20 in tris-buffered saline, TBS) at 4°C. The membranes were then probed with monoclonal mouse anti-human BORIS/CTCFL antibody (produced and kindly provided by Dr Dmitri Loukinov, NIH/NIAD) used at 1:1000 dilution in 1% blocking buffer (1% low-fat dried milk in TBS-T buffer) and incubated at room temperature for 1.5 hours. As loading control, mouse anti-human β-actin antibody (Sigma) at 1:5000 dilution in 1% blocking buffer was used and incubated at room temperature for 45 min. The membranes were washed 3× with TBS-T and incubated at room temperature for 1 hour with horseradish peroxidase (HRP)-labeled rabbit anti-mouse IgG (Sigma) diluted at 1:5000 in 5% blocking buffer. After 3× washing with TBS-T, the membranes were developed using WesternBright Quantum (Advansta) and visualized with Fusion FX Chemiluminescence System (Vilber Lourmat).

### Senescence-associated β-Galactosidase staining

Senescence-associated β-galactosidase (SA-β-gal) staining was performed using β-galactosidase staining kit (BioVision), according to the manufacturer's instructions. Briefly, 5×10^4^ cells/well were seeded in 12-well/plates in presence of doxycycline and were grown until confluence (5–7 days). Then, cells were rinsed with PBS, fixed for 15 min and incubated with freshly prepared SA-β-Gal staining solution at 37°C for 24 hour. After washing with PBS, SA-β-gal activity was observed using inverted microscope (Nikon) by detection of blue stained cells. At least 10 separate fields were selected. For each field the number of blue stained cells and the number of total cells were counted. Results are expressed as percentage of SA-β-gal-positive cells calculated as: (number of blue cells/number of total cells) x 100.

### Telomerase activity

Telomerase activity was measured using TRAPEZE RT telomerase detection kit (Millipore). This assay quantifies telomerase activity by SYBR Green real-time quantitative PCR [26]. Briefly, cells were lysed in 200 µl of CHAPS buffer and protein concentrations were determined with Nanodrop 2000. Aliquots of cell lysate (1.5 µg of protein/sample) were used. Inactivated samples, no-template reactions, and positive control were also assayed for quality control. A standard curve was prepared by serial dilution of TSR8 control template following manufacturer's instructions. Real-time amplifications were performed using Platinum Taq DNA Polymerase (2 unit/sample, Invitrogen). Cycling and fluorescence acquisition were done in Rotor Gene 6000 real-time PCR system (Corbett Life Science). Telomerase activity was calculated by comparing the average Ct values from each sample against the standard curve generated by the TSR8 control template.

### Statistical analysis

Statistical significance was evaluated using two-tailed student t-test analysis. P-value <0.05 was considered statistically significant.

## Results

### *In vitro* validation of the molecular beacons (MBs)

Two different MBs specific to *BORIS* mRNA (BORIS-MB1 and BORIS-MB2) and a RANDOM-MB were used in this experiment. The hybridization temperatures and specificity of the MBs were first tested in solution. The fluorescence emission of these MBs was monitored at temperatures ranging from 25 to 80°C, under different conditions: MB alone, MB in presence of the specific target, in presence of a non-specific target or in presence of a plasmid containing BORIS cDNA (pCMV-BORIS). As shown in Figure S1, noticeable fluorescence signal was detected only when the MBs were mixed with their specific targets. Optimal fluorescence emission with acceptable signal-to-background ratio (>4) was observed below 40°C. The assay also showed that BORIS-MBs discriminate single and double stranded structures, since they do not emit fluorescence when incubated with the pCMV-BORIS plasmid. These results demonstrated that MBs specifically hybridize to their target sequences and strongly suggested that these MBs would be able to specifically bind mRNA and not genomic DNA.

### Detection of *BORIS* mRNA using BORIS-MB

**Figure 1:**
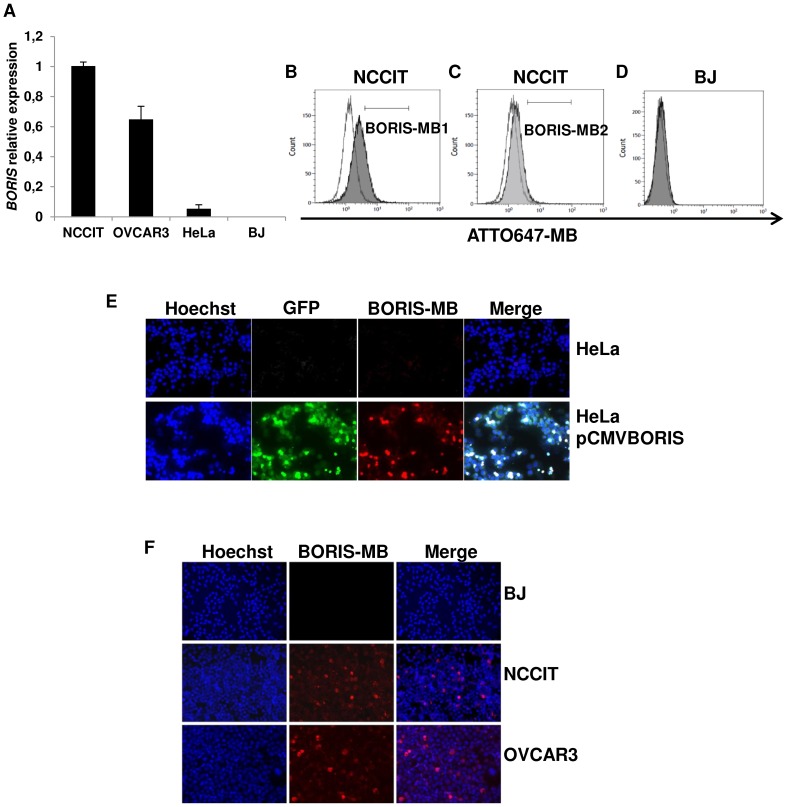
Detection of *BORIS* mRNA using BORIS-MB. (A) *BORIS* expression in human cell lines. Total RNA were isolated from human tumoral cell lines: NCCIT (embryonic), OVCAR3 (ovarian), HeLa (cervical) and normal BJ (fibroblast) cells. mRNA levels of *BORIS* were analyzed by qRT-PCR. Results were normalized to *GAPDH* and related to NCCIT cells. BJ and NCCIT were considered as negative and positive controls, respectively. Error bars represent the mean ± SD of 3 independent experiments. (B) Fluorescent signals measured by flow cytometry of NCCIT cells transfected with ATTO647-BORIS-MB1 (dark grey peak) and with ATTO647-RANDOM-MB (white peak). (C) Fluorescent signals measured by flow cytometry of NCCIT cells transfected with ATTO647-BORIS-MB2 (weak grey peak) and with ATTO647-RANDOM-MB (white peak). (D) Fluorescent signals measured by flow cytometry of BJ cells treated with ATTO647-BORIS-MB1 (from here onward referred to BORIS-MB, grey peak) and with ATTO647-RANDOM-MB (white peak). (E) *BORIS* expression in HeLa cells using BORIS-MB. Representative images of HeLa cells transiently transfected with the BORIS expression vector, pCMV-BORIS (bottom) and non-transfected control cells (top), 20× magnification. (F) *BORIS* expression in human cell lines as detected using BORIS-MB. Representative images of BJ, NCCIT and OVCAR3 cells, 20× magnification. For fluorescence imaging, 1×10^6^ cells were incubated at 37°C for 1 hour in serum-free DMEM medium with Cy3-BORIS-MB (200 nM). Hoechst 33342 5 µg/mL was added during the last 10 min of incubation. Slides were analyzed by fluorescence microscopy.

We first verified whether BORIS-MBs could be able to distinguish positive and negative BORIS expressing cells in living cells. Quantitative RT-PCR detected strong levels of *BORIS* mRNA in NCCIT and OVCAR3 cell lines, whereas this level was very low in HeLa cells and not detectable in BJ cells (Figure 1A), in accordance to previous studies [12]. Therefore, to study the specificity of MBs, NCCIT was used as a positive control cells and BJ as a negative control. NCCIT cells were transfected with BORIS-MB1 or BORIS-MB2 and fluorescence emissions were measured by flow cytometry. The mean fluorescent signals of the cells transfected with BORIS-MBs were higher compared to the mean fluorescent signal of the cells transfected with RANDOM-MB. An increase of 23.2 and 4.7 was observed for BORIS-MB1 and BORIS-MB2, respectively (Figure 1B–C). However, since BORIS-MB1 provided higher (5 fold) mean fluorescence signal compared to BORIS-MB2, BORIS-MB1 was selected for the subsequent analysis. From here onward BORIS-MB1 is referred as BORIS-MB. As expected, when BORIS negative BJ cells were transfected with BORIS-MB, they did not show any fluorescence signal (Figure 1D).

To further challenge the specificity of the BORIS-MB, HeLa cells that expressed *BORIS* mRNA at low level, were transiently transfected with the pCMV-BORIS expression plasmid, which contains the human BORIS cDNA fused to green fluorescent protein (GFP) gene. As expected, the transfected cells presented high fluorescence signals of both GFP and BORIS (Figure 1E). All together, these results confirmed the capacity of the BORIS-MB to reliably and specifically detect *BORIS* mRNA in living cells.

Hence, the cell lines were transfected with the BORIS-MB to visualize *BORIS* mRNA expression by fluorescence imaging. As can be seen from Figure 1F, the fluorescence signal of BORIS-MB was clearly visible in NCCIT and OVCAR3 cells, in which only a subset of cells were fluorescent. The percentage of BORIS positive cells in these cell lines was found to be between 3 and 5%. As expected, in BJ normal cell line no fluorescent cells were observed. In HeLa cells, the number of BORIS positive cells was extremely low, less than 0.1% (Figure 1E). Overall these experiments demonstrated that within tumor cell lines, *BORIS* mRNA is not present in all cells but rather occurs only in a subset of cells.

### Isolation of cell population expressing *BORIS* mRNA using BORIS-MB

**Figure 2:**
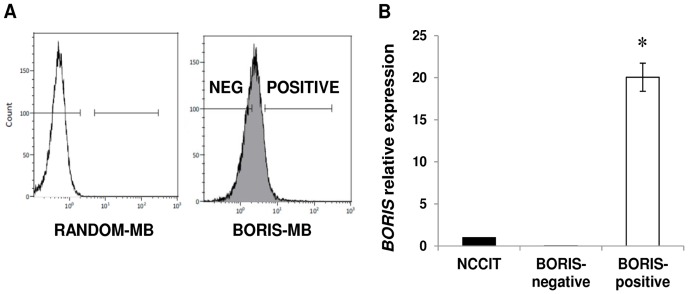
Isolation of BORIS-positive cells using BORIS-MB. (A) NCCIT cells were transfected with ATTO647-RANDOM-MB (white peak) and ATTO647-BORIS-MB (grey peak). The two subpopulations, BORIS-negative and BORIS-positive cells were selected by comparing the fluorescent signal of RANDOM-MB to that of BORIS-MB. After exclusion of dead cells by PI staining, the two fractions were sorted. (B) *BORIS* expression of the isolated BORIS-negative and BORIS-positive fractions was analyzed by qRT-PCR. The results were normalized to *GAPDH* and related to non-sorted NCCIT cells. Error bars represent the mean ± SD of 3 independent experiments. Asterisk indicates statistically significant difference (p<0.05) between BORIS-positive and non-sorted cells.

NCCIT cell line was used for the isolation of BORIS-positive cells. FACS sorting was performed after cell transfection with BORIS-MB. The BORIS-positive and BORIS-negative cells (8.4±1.5 and 41.5±7.2% of total, respectively; mean ± SD) were sorted (Figure 2A).

*BORIS* expression analysis showed that *BORIS* mRNA level of sorted BORIS-positive cells was 20 fold higher compared to the non-sorted cells (Figure 2B). This result demonstrated the efficiency of the sorting method and the successful enrichment of a cell population that expressed *BORIS* mRNA.

### The isolated BORIS-positive and BORIS-negative cells have similar methylation pattern of BORIS promoter B

**Figure 3:**
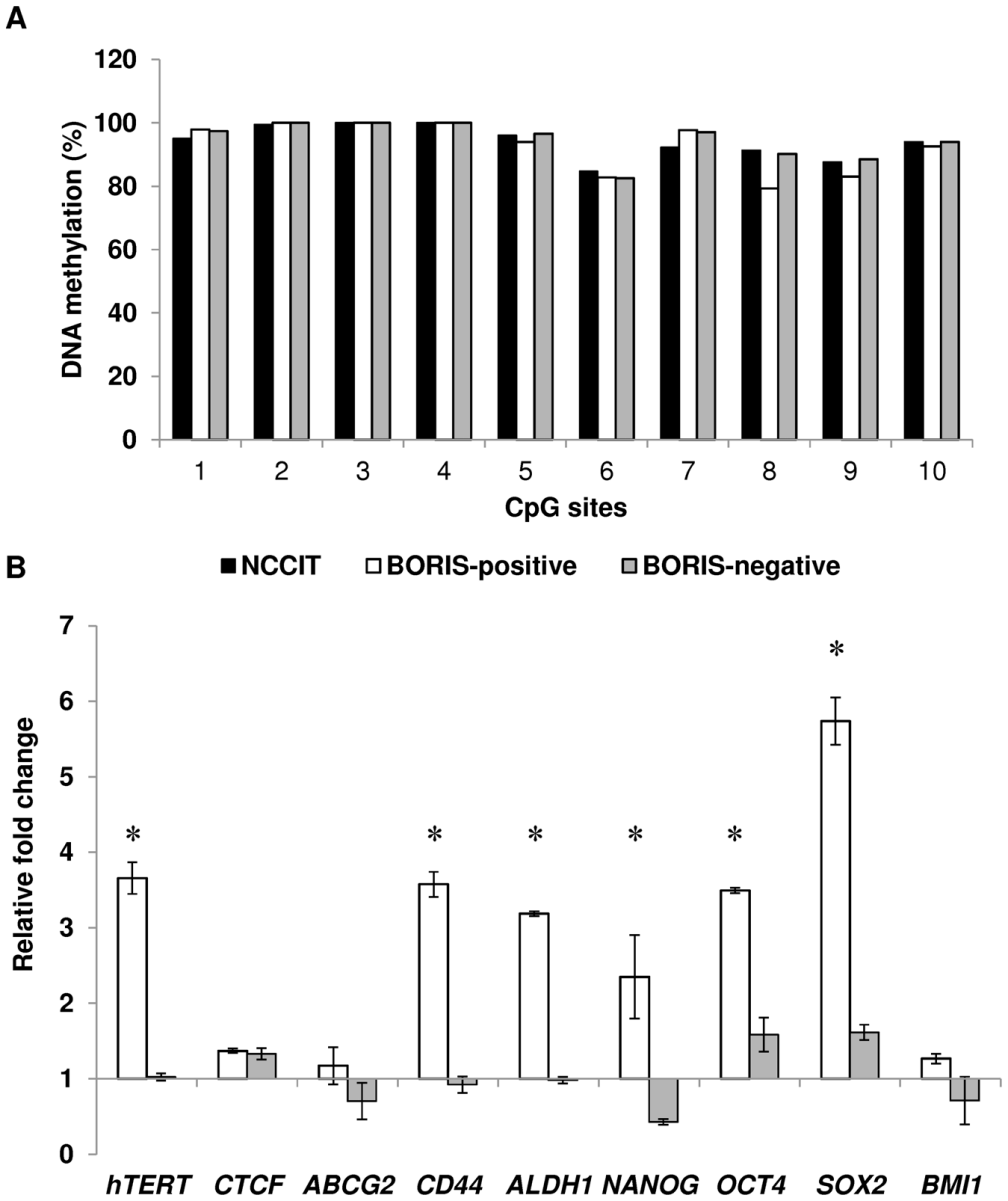
BORIS promoter methylation and expression of *hTERT*, stem cell and CSC markers genes in BORIS-positive cells. (A) Methylation analysis of 10 CpG islands within the *BORIS* promoter region (B promoter). The representative graphic shows the percentage of methylation of each CpG island for the isolated BORIS-positive (white), -negative (grey) and non-sorted (black) NCCIT cells. (B) Expression analysis of the isolated BORIS-positive (white) and BORIS-negative (grey) fractions. The indicated genes were analyzed by qRT-PCR. The results were normalized to *GAPDH* and related to non-sorted NCCIT cells. Graphic shown one representative experiment out of 3 independent experiments (the trend was similar in all independent experiments). Asterisks indicate statistically significant difference (p<0.05) between BORIS-positive and the non-sorted cells.

In a previous study, we have shown that *BORIS* expression is controlled by three alternative promoters, corresponding to transcription start sites at −1447, −899 and −658 bp upstream of the first ATG and designated promoters as A, B and C, respectively [25]. Interestingly, it has been observed that in tumors, demethylation of BORIS promoter B, is generally correlated with the expression of *BORIS*, which is not the case for promoters C and A [25]. Consequently, we interrogated the possible correlation between methylation of promoter B and *BORIS* expression in the sorted cells. BORIS methylation level of this promoter was measured by pyrosequencing, after bisulfite modification of DNA extracted from BORIS-positive and BORIS-negative cells. Pyrosequencing results indicated that in both fractions, the CpGs were heavily methylated (85–100% methylation) and no differences were detected (Figure 3A). This confirmed that in NCCIT cells, *BORIS* expression was not regulated by promoter B and suggested that the different expression of *BORIS* among the sorted fractions was not guided by DNA methylation status of promoter B but rather involved other levels of control.

### BORIS-positive cells express higher mRNA levels of *hTERT* as well as stem cell and cancer stem cell markers genes

We previously established that BORIS binds *hTERT* promoter at the same site of CTCF and can activate *hTERT* transcription in NCCIT cells [12]. To further investigate the correlation between *BORIS*, *hTERT* and *CTCF* expression, BORIS-positive and BORIS-negative populations were sorted as mentioned above and the expression of these genes was evaluated by qRT-PCR. Interestingly, three independent sorting experiments showed that *hTERT* mRNA level was significant higher (from 3.5 to 3.8 fold) in the BORIS-positive population compared to the BORIS-negative population. This result confirmed the positive correlation between *BORIS* and *hTERT* expression, whereas no difference was observed for *CTCF* (Figure 3B).

Expression of *hTERT* has been frequently associated with expression of stemness-related markers [13]-[17]. Consequently, considering our results, we also investigated if this correlation could exist with *BORIS* expression. To assess this, a panel of representative genes considered as stemness markers (*NANOG, OCT4, SOX2* and *BMI1*) or as specific cancer stem cell (CSC) markers (*ABCG2, CD44* and *ALDH1*) [27] were analyzed by qRT-PCR. Interestingly, it emerged that the BORIS-positive/hTERT-high population also expressed higher levels of stem cell-like markers. Indeed, mRNA levels of mostly all these genes were significantly higher in BORIS-positive population compared to BORIS-negative population and non-sorted cells (between 2 and 6 fold in average), at exception of *BMI1* and *ABCG2* genes (Figure 3B).

### Knockdown of *BORIS* down-regulates expression of *CTCF*, *hTERT*, stem cell and CSC markers genes

**Figure 4:**
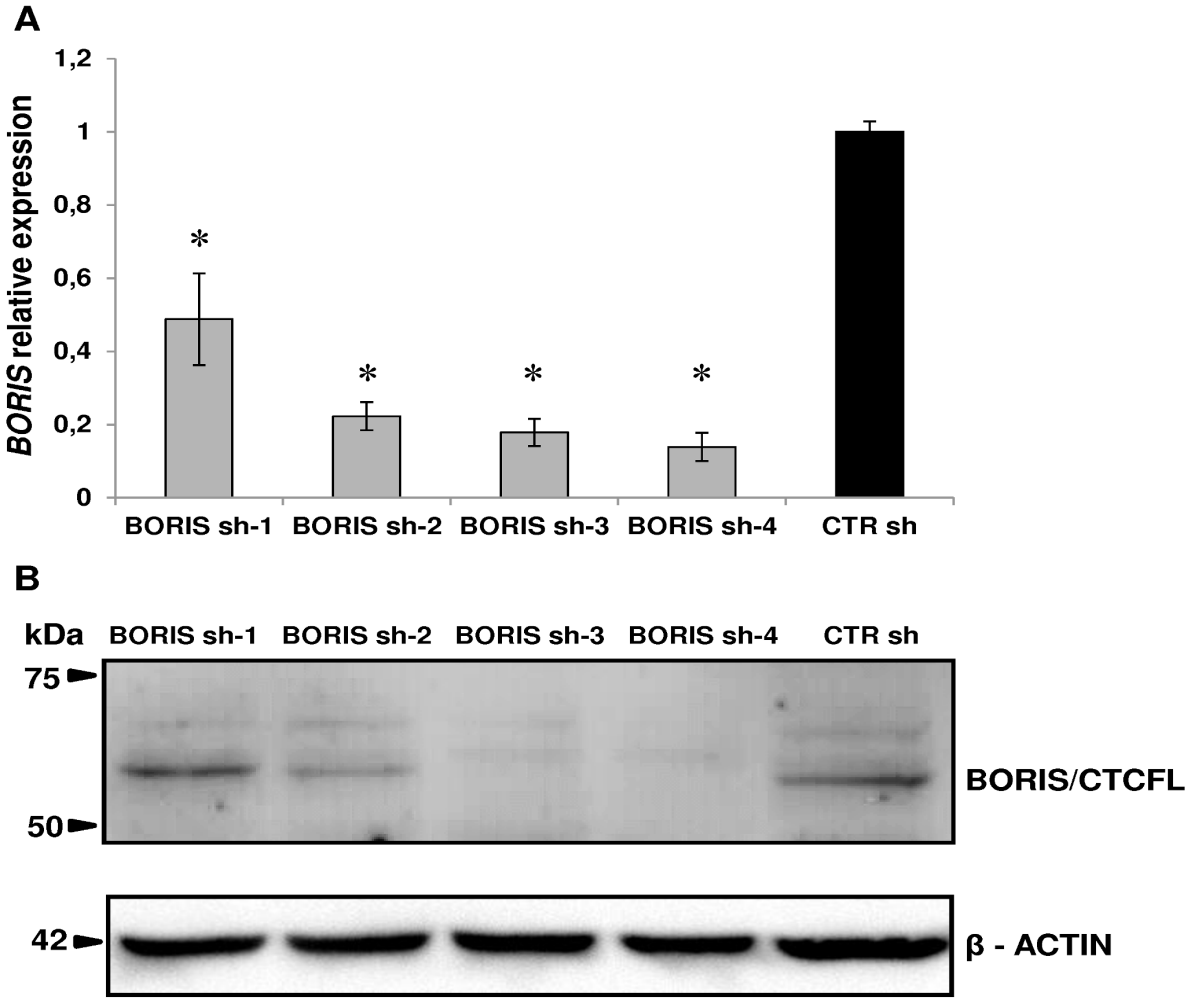
*BORIS*-knockdown using lentivirus with doxycycline inducible BORIS-specific shRNA. Four different BORIS shRNA lentiviral vectors (BORIS sh-1, sh-2, sh-3 and sh-4) and the vector carrying a scrambled sequence (CTR sh) were tested. NCCIT were transduced with the indicated lentivirus and sorted for eGFP marker expression. Then, cells were cultured with doxycycline-containing medium and after 3 days were analyzed. (A) *BORIS* mRNA levels were determined by qRT-PCR analysis, normalized to *GAPDH* and compared to that of CTR sh. Error bars represent the mean ± SD of 3 independent experiments. Asterisks indicate statistically significant difference (p<0.05) between BORIS sh-1, sh-2, sh-3 or sh-4 and CTR sh cells. (B) Representative western blot analysis. BORIS and β-actin (as a loading control) protein levels were determined by western blot. For both analyses (qRT-PCR and western blot) the knockdown was especially noticed with the BORIS sh-3 and sh-4 compared to the CTR shRNA.

**Figure 5:**
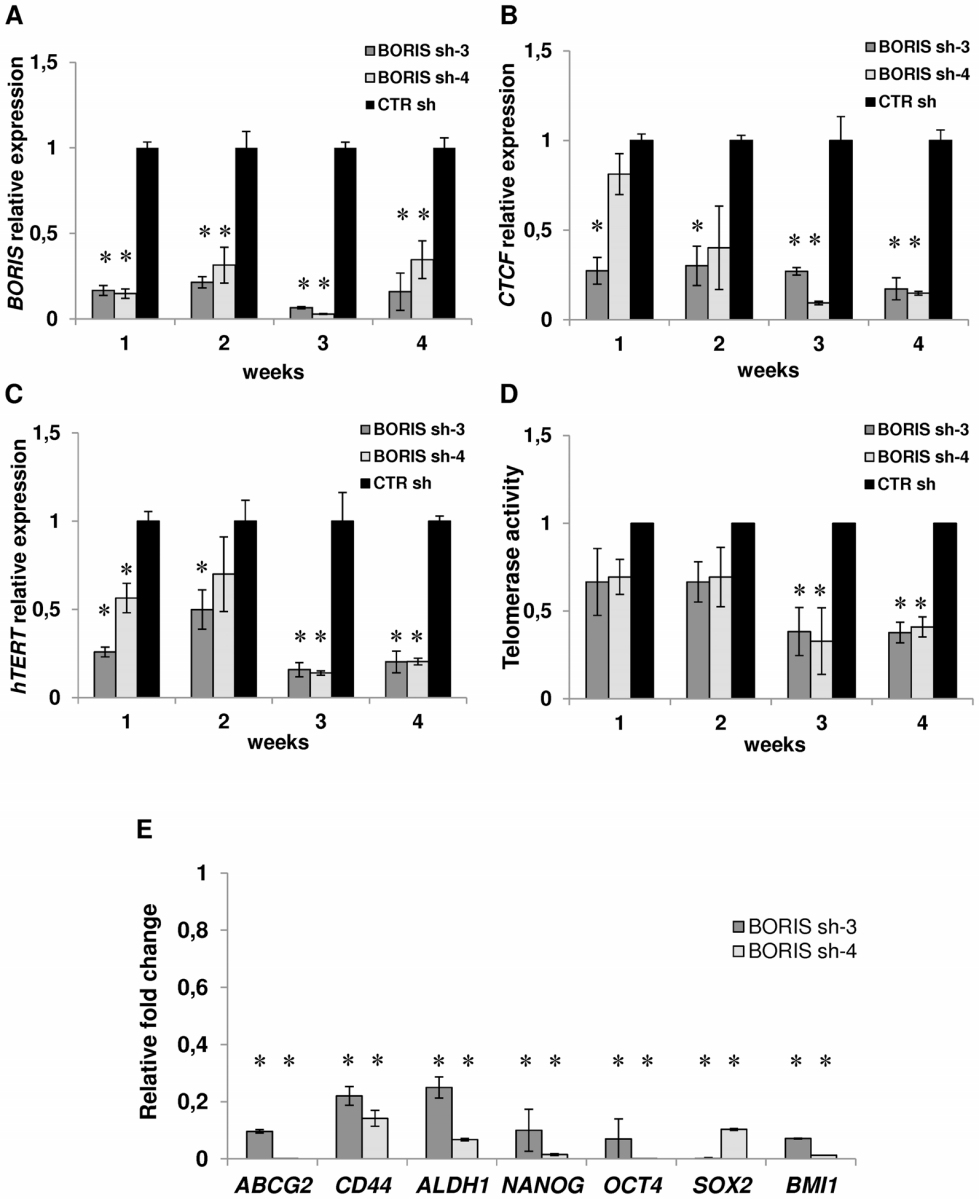
Knockdown of *BORIS* resulted in decreasing expression of *hTERT*, stem cell and CSC markers genes. NCCIT cells were engineered to stably exhibit knocked-down *BORIS* mRNA. BORIS sh-3, sh-4 and CTR sh (control with scrambled sequence) lentivirus were used to infect NCCIT cells. Each transduced cells were cultured with doxycycline to induce BORIS shRNA expression. Doxycycline-containing medium was replaced every 3 days. Each week over 1 month, mRNA levels of (A) *BORIS*, (B) *CTCF* and (C) *hTERT* were analyzed by qRT-PCR. Results were normalized with *GAPDH* and related to that of control cells (CTR sh) at each week. Error bars represent the mean ± SD of 3 independent experiments. (D) Telomerase activity was measured at each week by real-time quantitative PCR using TRAPEZE RT Telomerase Detection Kit. Values of telomerase activity of BORIS sh-3, sh-4 NCCIT-derived cells are related to that of control cells at each week. Error bars represent the mean ± SD of 3 independent experiments. (E) BORIS sh-3, sh-4 and CTR sh NCCIT-derived cells were cultured with doxycycline and after 7 days RNA was analyzed by qRT-PCR. mRNA levels of the indicated genes are related to that of control cells (CTR sh) after normalization with *GAPDH*. Error bars represent the mean ± SD of 3 independent experiments. Asterisks indicate statistically significant difference (p<0.05) between BORIS sh-3 or BORIS sh-4 and CTR sh cells.

To investigate more directly the functions of BORIS, stable cell lines with inducible expressing shRNAs, targeting human *BORIS* mRNA, were generated using a lentiviral system. Four different BORIS shRNA lentivirus (BORIS sh-1, sh-2, sh-3 and sh-4) and a lentivirus carrying a scrambled sequence (CTR sh) were produced and tested. A significant reduction of *BORIS* mRNA was observed in all BORIS-depleted NCCIT-derived cells, compared to control cells (Figure 4A). The western-blot analysis indicated a remarkable decrease of BORIS protein level in BORIS sh-3 and sh-4 cells (Figure 4B). Therefore, all following knockdown experiments were performed using these BORIS sh-3 and sh-4 NCCIT-derived cells. The capacity of doxycycline-induction to maintain *BORIS*-knockdown overtime was also verified. As shown in figure 5A, *BORIS* mRNA levels were significantly reduced during 1 month. Interestingly, *CTCF* expression was dramatically decreased (Figure 5B). As expected, *hTERT* expression was down-regulated compared to control (Figure 5C) and this down-regulation was even stronger after 3 weeks. This observation was consistent with the correlation observed in the sorting experiments (Figure 3A) and was further confirmed by telomerase activity analysis. Indeed, we observed that telomerase activity was also decreased, especially after 3 and 4 weeks of *BORIS* silencing (Figure 5D). Notably, absence of *BORIS* triggered a dramatic down-regulation (between 75% and 99%) of the expression of stem cell and CSC marker genes (Figure 5E). This down-regulation was consistent up to the third week (data not shown).

All these results confirmed that BORIS regulates *hTERT* and strongly suggested that BORIS affects the transcription of stem cell and CSC marker genes, a pattern of genes expressed in CSCs.

### Knockdown of *BORIS* inhibits cell proliferation through cellular senescence

**Figure 6:**
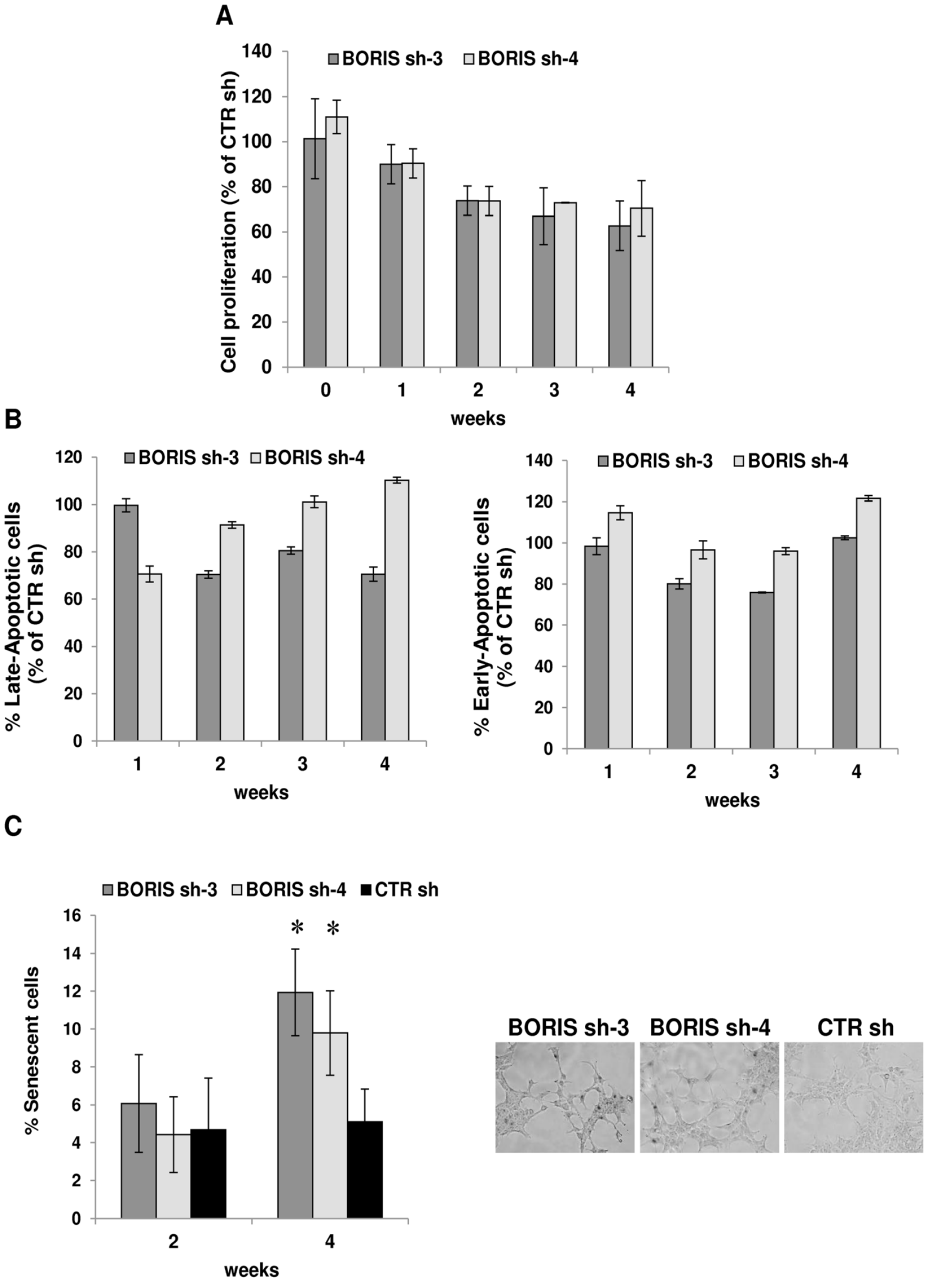
*BORIS* knockdown impairs cell senescence but not apoptosis. (A) Cell proliferation over 1 moth of dox-induced BORIS shRNA cells were analyzed by MTT assay. Results of the two specific BORIS-shRNA (BORIS sh-3 and sh-4) NCCIT-derived cells are indicated as a percentage compared to the cell proliferation of control cells (scrambled shRNA, CTR sh). Error bars represent the mean ± SD of 3 independent experiments. (B) After dox-induction of the BORIS specific shRNA in NCCIT cells, apoptosis was tested at each week using Annexin V Apoptosis Detection Kit. Results show the percentage of apoptotic cells (late apoptotic AnnexinV^+^/7AAD^+^ and early apoptotic AnnexinV^+^/7AAD^−^) of BORIS sh-3 and sh-4 cells compared to the control cells. Error bars represent the mean ± SD of 3 experiments. (C) The senescence-associated β-galactosidase (SA-β-gal) staining was performed using β-galactosidase staining kit. SA-β-gal was analyzed after 2 and 4 weeks of dox-induction of the BORIS specific shRNA in NCCIT cells. Results show the percentage of senescent cells of BORIS sh-3, BORIS sh4 and control cells. Error bars represent the mean ± SD of 3 experiments. Asterisks indicate statistically significant difference (p<0.05) between BORIS sh-3 or BORIS sh-4 and CTR sh cells. Representative images of NCCIT cells after 4 weeks of *BORIS* knockdown and stained with SA-β-gal were shown.

The impact of *BORIS* silencing on cell survival of embryonic tumor cells was also investigated. Cell proliferation was measured each week during 1 month by MTT assay (Figure 6A). An inhibition of cell growth was observed starting from the second week with 26% of reduction, to the fourth week with 40% of decrease compared to control. The analysis of apoptosis showed that the percentage of apoptotic cells (late apoptotic AnnexinV^+^/7AAD^+^ and early apoptotic AnnexinV^+^/7AAD^−^) was not significantly different between BORIS knockdown cells and control cells (Figure 6B). This result suggested that the decreasing of the observed cell growth is not due to cell apoptosis, therefore we further explored the causes of proliferation defects. As *BORIS*-knockdown led to a reduction of *hTERT* expression, a possible alteration of cellular senescence was investigated. Interestingly, analysis of senescence-associated β-galactosidase showed that, after 4 weeks of *BORIS*-knockdown, the percentage of senescent cells was 2 fold higher in BORIS silenced cells compared to control cells (Figure 6C). The percentage of senescent cells increased from 5.1 ± 1.7% in control cells to 11.9±2.3% and 9.8±2.2% in BORIS silenced with sh-3 and sh-4, respectively. These results indicated that BORIS knockdown led to the increase of cellular senescence in embryonic cancer cells.

## Discussion

Our study showed that in embryonic tumor cell lines, BORIS positive cells represent only a subset of the tumor cell population and the estimated frequency is approximately 3–5% of the total cells. This observation was confirmed by performing experiments with *BORIS* mRNA-targeting MB. Molecular beacon technology provides a powerful tool to discriminate target sequences, with very high specificity. Since their discovery [28], the MBs have been seen to distinguish target sequences differing only by a single nucleotide. Due to the characteristics of exceptional specificity and high sensibility, MBs have found a wide range of applications in biological sciences. MBs were used as taqman probes in real time quantitative PCR, for detection of mutations, SNP and allele, as indicators of contaminating infectious agents and also for *in vivo* detection of mRNA [29]. Furthermore, MBs are able to bind target sequences without modifying the functional and genomic characteristics of the cells. To date, there is no yet a validated antibody for *in vivo* detection of BORIS, therefore we used the MB technology to visualize *BORIS* mRNA positive cells. Recently, two publications demonstrated the use of MBs in FACS sorting [30], [31]. However, they showed different delivery systems of MBs to enter into the cells. One group described a delivery system with a cationic lipid vehicle [30] and the other group used electroporation with a dual-FRET MB [31]. Instead, we used the RNAiMAX Transfection Reagent, a cationic lipid formulation that was designed specifically for delivery single strand nucleotides (siRNA and miRNA). Additionally, we used for the sorting experiments, BORIS-MB conjugated with ATTO647, a fluorophore which confers a high photostability of fluorescence signal [20]. The analysis of *BORIS* expression demonstrated that we successfully enriched the cell population that expressed *BORIS* mRNA. In our experiments we used NCCIT cell line, classified as germ-cell tumor or embryonic cancer cells [32]. Due to its higher *BORIS* expression compared to the other tumor cell lines, the NCCIT cell line provides a good model for our studies, especially for the feasibility of isolation of BORIS positive cells by MB technology.

In a previous study, we have shown that BORIS modulates the transcription of *hTERT* telomerase gene in NCCIT and OVCAR3 cells, and in opposite manner compared to its paralog CTCF [12]. To further investigate the correlation between *BORIS, hTERT* and *CTCF*, we analyzed their expression in FACS-isolated BORIS-positive and BORIS-negative cells. The results confirmed the positive correlation between *BORIS* and *hTERT*, whereas no correlation was observed with *CTCF*. In the same previous work, it has been found that ectopic *BORIS* expression in normal BORIS negative cells allowed to expand the *in vitro* lifespan increasing cell passages. This finding could be explained by the expression of high levels of *hTERT* mRNA in these BORIS-transfected cells. Induction of *hTERT* expression and telomerase activity are well established as hallmarks of cancer and are prerequisite to cellular immortalization and malignant transformation [33]. Here, we confirm the role of BORIS in the immortalization process during tumorigenesis, since the isolated BORIS-positive cells expressed significantly higher levels of *hTERT* mRNA compared to the counterpart BORIS-negative cells.

In normal human cells, telomerase is generally absent in somatic cells but remains active in germ cells, progenitor cells and some adult stem cells [14]. It has been shown that telomerase is reactivated in a majority (approximately 90%) of tumors [34] and in clinical studies its reactivation is associated with poor outcomes of different tumors [35]–[38]. In addition, current literature supports the evidence that CSCs express telomerase and its inhibition suppresses the self-renewal of CSCs [39]–[41]. All these discoveries, together with our observations, indicate that BORIS could play an important and direct role in tumor malignancies by up-regulation of the *hTERT* telomerase gene. Consistent with this, we notably observed that the BORIS-positive/hTERT-high isolated cells expressed also high levels of the most important stem cell markers. The embryonic carcinoma cells that we investigated, indeed, provide a good model system to study the stem cell concept of cancer. They are stem cells derived from a teratocarcinoma and are also the malignant transformed embryonic stem cells [42]. They show gene expression profiles close to those of human embryonic stem cells [43]. Therefore, we analyzed the association of BORIS-positive/hTERT-high cells with the expression of the key-regulator genes of embryonic cells (*NANOG, SOX2* and *OCT4*) and with some of the most known specific markers of CSCs [27]. CSCs are cancer cells characterized by stem cell properties and represent a small population of tumor cells that drives tumor development, progression, metastasis and drug resistance [27]. Interestingly, a correlation of the isolated BORIS-positive/hTERT-high cells with high expression of stem cell markers was observed. These findings were confirmed by *BORIS* silencing studies. Stable BORIS knockdown NCCIT-derived cells were generated by an efficient system of inducible-shRNA lentivirus [21]. After BORIS silencing, a significant decrease of *hTERT* expression was observed, as well as a down-regulation of telomerase activity, which is strictly regulated by *hTERT* gene transcription. *CTCF* expression was also decreased. This is consistent with our previous results [12] and confirms a role of BORIS in the transcriptional regulation of *CTCF*. This correlation between CTCF and BORIS was not observed in the expression analysis of the isolated BORIS-positive cells. This discrepancy could be due to the different experimental conditions. Indeed, in the expression analysis of the isolated BORIS-positive cells, *CTCF* was analyzed at steady state, while in *BORIS* silencing studies what we observed is the result of cellular and genetic modifications.

Importantly, after BORIS knockdown a decrease of expression of stem cell and CSC marker genes (*NANOG, OCT4, SOX2, BMI1, ABCG2, CD44* and *ALDH1*) was also observed. These results highlight the importance of BORIS in malignant disease and its possible critical role on cancer growth and progression. Previous works have already showed a correlation of BORIS with stem cells. Monk et al. have observed the co-localization at the protein level of BORIS with ECSA, OCT4 and NANOG in cultured embryonic stem cells [44]. John et al. have detected BORIS expression in ECSA-expressing lung tumors [45]. In this paper, we additionally showed the molecular function of BORIS in embryonic cancer cells and all these data strongly suggested that BORIS affects the expression of stem cell genes.

Cell proliferation analysis revealed that the depletion of BORIS led to cell growth inhibition and an increase of cellular senescence in embryonic cancer cells. Cellular senescence is defined as the irreversible arrest of cell growth that is activated after alteration of telomeres or in response to different forms of stress [46]. Of note, cellular senescence is considered as a potent tumor suppressive mechanism, a protective barrier against neoplastic expansion [47]. Senescent cells cannot divide, even if they continue to be metabolically and synthetically active [48]. Senescent cells also show changes in chromatin organization and gene expression [49]. In our studies, the cellular senescence was measure by the most widely used senescence associated marker, the â-galactosidase activity [50]. The increasing of senescent cells after BORIS knockdown could be caused by the simultaneous telomerase inhibition. Consistent with the finding that inhibition of telomerase has been shown to initiate telomere shortening followed by cell senescence and cell death by apoptosis [51], [52].

In summary, the present study provides evidences that BORIS is only expressed in a small subset of tumor cells in embryonic tumors. As BORIS affects the expression of stemness-related genes, this subpopulation of BORIS positive cells might play an important role in cancer growth and progression. Our findings provide a rationale for investigating therapies that target BORIS/CTCFL in embryonic tumors.

## Supporting Information

Figure S1 **Fluorescence emission profiles of MBs.** Representative fluorescence emission profiles of BORIS-MB1, BORIS-MB2 and RANDOM-MB. All the MBs were 5′-end Cy3-conjugated. All thermal profiles indicate the MBs (200 nM) alone (green dash line) and MBs mixed with specific target (red solid line), with non-specific target (black dash-dot line) and with plasmid (pCMV-BORIS, blue dot line). The targets were used at the final concentration of 1 µM. The samples were analyzed by Rotor Gene 6000 Real-Time PCR system and the fluorescence was measured at each temperature (from 25°C to 80°C) using filter for Cy3.
(DOCX)

Table S1 **Primer sequences for qRT-PCR analysis.**
(DOCX)
