# Supplementary material for: High Expression of hTERT and Stemness Genes in BORIS/CTCFL Positive Cells Isolated from Embryonic Cancer Cells
Source: PLoS One. 2014 Oct 3;9(10):e109921. doi: 10.1371/journal.pone.0109921 (PMC4184884; doi:10.1371/journal.pone.0109921)
Supplement: Figure S1 — Fluorescence emission profiles of MBs. Representative fluorescence emission profiles of BORIS-MB1, BORIS-MB2 and RANDOM-MB. All the MBs were 5′-end Cy3-conjugated. All thermal profiles indicate the MBs (200 nM) alone (green dash line) and MBs mixed with specific target (red solid line), with non-specific target (black dash-dot line) and with plasmid (pCMV-BORIS, blue dot line). The targets were used at the final concentration of 1 µM. The samples were analyzed by Rotor Gene 6000 Real-Time PCR system and the fluorescence was measured at each temperature (from 25°C to 80°C) using filter for Cy3. (DOCX) [file pone.0109921.s001.docx]

**
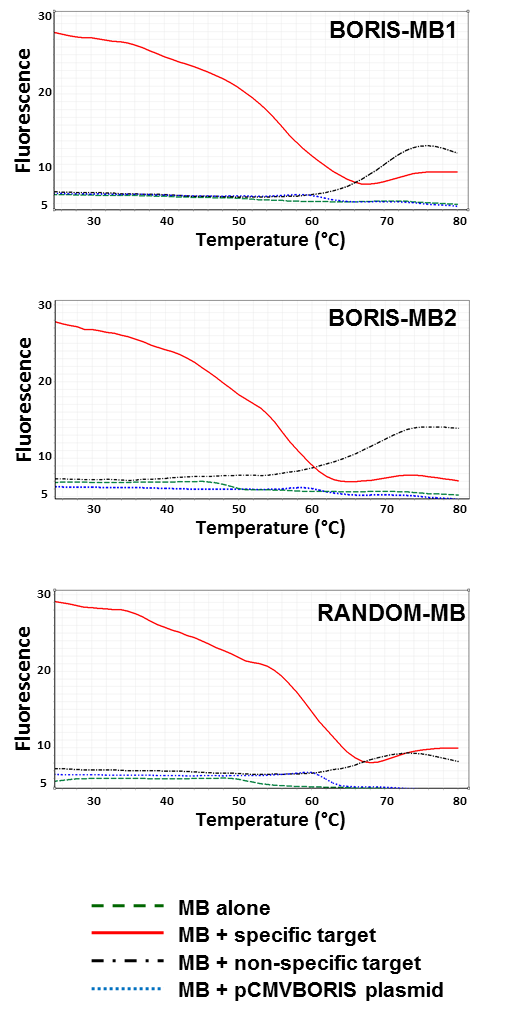
**

**Figure S1.** **Fluorescence emission profiles of MBs.** Representative fluorescence emission profiles of BORIS-MB1, BORIS-MB2 and RANDOM-MB. All the MBs were 5’-end Cy3-conjugated. All thermal profiles indicate the MBs (200 nM) alone (green dash line) and MBs mixed with specific target (red solid line), with non-specific target (black dash-dot line) and with plasmid (pCMV-BORIS, blue dot line). The targets were used at the final concentration of 1 µM. The samples were analyzed by Rotor Gene 6000 Real-Time PCR system and the fluorescence was measured at each temperature (from 25°C to 80°C) using filter for Cy3.
