# Supplementary material for: High Expression of hTERT and Stemness Genes in BORIS/CTCFL Positive Cells Isolated from Embryonic Cancer Cells
Source: PLoS One. 2014 Oct 3;9(10):e109921. doi: 10.1371/journal.pone.0109921 (PMC4184884; doi:10.1371/journal.pone.0109921)
Supplement: Table S1 — Primer sequences for qRT-PCR analysis. (DOCX) [file pone.0109921.s002.docx]

| **Gene**  **Table 1** **Primer sequences for qRT-PCR analysis** | **Forward primer** | **Reverse primer** |
| --- | --- | --- |
| BORIS | 5' GCCCTCATTCAGCACCAGAAAAC 3' | 5' CTCCAGTGTGGGTACGAATGTGA 3' |
| CTCF | 5’ GTGGCAGGGCATTCAGAACAG 3’ | 5’ CGATGCCGAACCAATTCTCCAC 3’ |
| hTERT | 5’ TGACACCTCACCTCACCCAC 3’ | 5’ CACTGTCTTCCGCAAGTTCAC 3’ |
| ALDH1 | 5’ GCAACTGAGGAGGAGCTCTG 3’ | 5’ AAGCATCCATAGTACGCCAC 3’ |
| BMI1 | 5’ GCTAAATCCCCACCTGATGT 3’ | 5’ GGTCTCCAGGTAACGAACAA 3’ |
| NANOG | 5’ ATACCTCAGACTCCAGCAGA 3’ | 5’ TCTGGAACCAGGTCTTCACC 3’ |
| OCT4 | 5’ GGTATTCAGCCAAACGACCA 3’ | 5’ TTCTCTTTCGGGCCTGCACG 3’ |
| SOX2 | 5’ CCTGGCATGGCTCTTGGCTC 3’ | 5’ TGGAGTGGGAGGAAGAGGTA 3’ |
| CD44 | 5’ TAAGGACACCCCAAATTCCA 3’ | 5’ ACTGCAATGCAAACTGCAAG 3’ |
| GAPDH | 5’ AAGGTGAAGGTCGGAGTCAAC 3’ | 5’ GAGTTAAAAGCAGCCCTGGTG 3’ |

**Table S1.** **Primer sequences for qRT-PCR analysis**
